# Supplementary material for: The association between the error-related negativity and self-control is moderated by impulsivity and compulsivity
Source: Commun Psychol. 2026 Mar 27;4:62. doi: 10.1038/s44271-026-00446-3 (PMC13046855; doi:10.1038/s44271-026-00446-3)
Supplement: Supplementary file 2 — Supplementary Information [file 44271_2026_446_MOESM2_ESM.pdf]

Supplementary Material

Title: The association between the error-related negativity and self-control is moderated by impulsivity and compulsivity

Authors: Rebecca Overmeyer[1]*, Anja Kräplin[2], Thomas Goschke[1,3] & Tanja Endrass[1,3]

1 Faculty of Psychology, Dresden University of Technology, Dresden, Germany
2 Department of Psychiatry and Psychotherapy, Dresden University of Technology, Germany
3 Neuroimaging Centre, Dresden University of Technology, Dresden, Germany

Correspondence should be addressed to Rebecca Overmeyer (email: rebecca.overmeyer@tu-dresden.de)

Supplementary Notes 1: Sample characteristics 2

Supplementary Notes 2: The effects of impulsivity and compulsivity on desire enactment 7

Supplementary Notes 3: The effects of impulsivity and compulsivity on the association between the ERN and desire enactment 9

Supplementary Notes 4: Additional analyses regarding the correct-related negativity and behavioral parameters 18

Supplementary Notes 5: Descriptive statistics on desire type and conflict 19

Supplementary Notes 6: Additional analyses for the Profile regression 19

Supplementary Notes 1: Sample characteristics

1.1 Sample characteristics. See Supplementary Table 1 for psychopathological symptoms and personality traits sample characteristics, Supplementary Figure 1 for correlations between these measures, Supplementary Table 2 for measures of performance monitoring and task performance, Supplementary Figure 2 for a depiction of the error-related negativity (ERN) and the correct-related negativity (CRN), and Supplementary Table 3 for task characteristics regarding trial type for the monetary incentive flanker task.

1.2 Psychopathological symptoms and personality traits potentially related to goal-directed control assessed in the laboratory. *Impulsivity* was assessed using the 11th version of the Barratt Impulsiveness Scale (BIS-11) [1-3] which is conceptualized in terms of attentional, motor and non-planning impulsivity facets. The total score yields good internal consistency (α = .83)[2]. *Compulsivity* was assessed using the Obsessive-Compulsive Inventory-Revised (OCI-R) [4,5]. The total score determines the severity of obsessive-compulsive symptoms (including washing, checking, doubting, ordering, obsessing, hoarding, and neutralizing) and yields good internal consistency (α = .85) [5].
Additional impulsivity facets were assessed using the Urgency, Premeditation (lack of), Perseverance (lack of), Sensation Seeking, Impulsive Behavior Scale [6-8]. Depression, anxiety and worry were measured using the Depression Anxiety Stress Scales [9,10], the State-Trait Inventory for Cognitive and Somatic Anxiety [11,12], and the Penn State Worry Questionnaire [13,14]. Habitual propensity was operationalized using the Creature of Habit Scale [15,16]. Behavioral inhibition and activation were measured using the BIS/BAS scales [17,18].

Supplementary Table 1. Sample characteristics derived from questionnaire data

	Mean	*SD*	*Range*
Impulsivity	60.57	8.95	38 - 96
Compulsivity	12.74	9.33	0 - 46
Urgency	26.31	5.86	14 - 43
Lack of premeditation	22.38	4.36	13 - 38
Lack of perseverance	19.38	4.29	10 - 31
Sensation seeking	33.05	7.04	14 - 46
Depression (DASS-21)	2.76	2.94	0 - 19
Anxiety (DASS-21)	1.89	2.57	0 - 20
Somatic anxiety	14.40	2.84	11 - 26
Cognitive anxiety	15.81	3.98	10 - 32
Worry	43.22	11.39	22 - 74
Automaticity	29.80	7.79	11 - 51
Routine	51.23	9.24	27 - 75
Behavioral inhibition	20.29	3.68	10 - 28
Behavioral activation	40.39	4.56	26 - 52

Impulsivity and compulsivity: Barratt Impulsiveness Scale, revised [1]; Obsessive-Compulsive Inventory-Revised [4,5]; additional impulsivity facets: Urgency, Lack of premeditation, Lack of perseverance, Sensation seeking, Impulsive Behavior Scale [6-8]; Depression (DASS-21) and Anxiety (DASS-21): Depression Anxiety Stress Scales [9]; Somatic and Cognitive anxiety: State-Trait Inventory for Cognitive and Somatic Anxiety [11]; Rumination: Penn State Worry Questionnaire [13]; Automaticity and Routine: subscales of habitual propensity measure Creature of Habit Scale [15,16]; Behavioral inhibition and activation: BIS/BAS scales [17,18].

Supplementary Figure 1: Correlations between sample questionnaire scores.

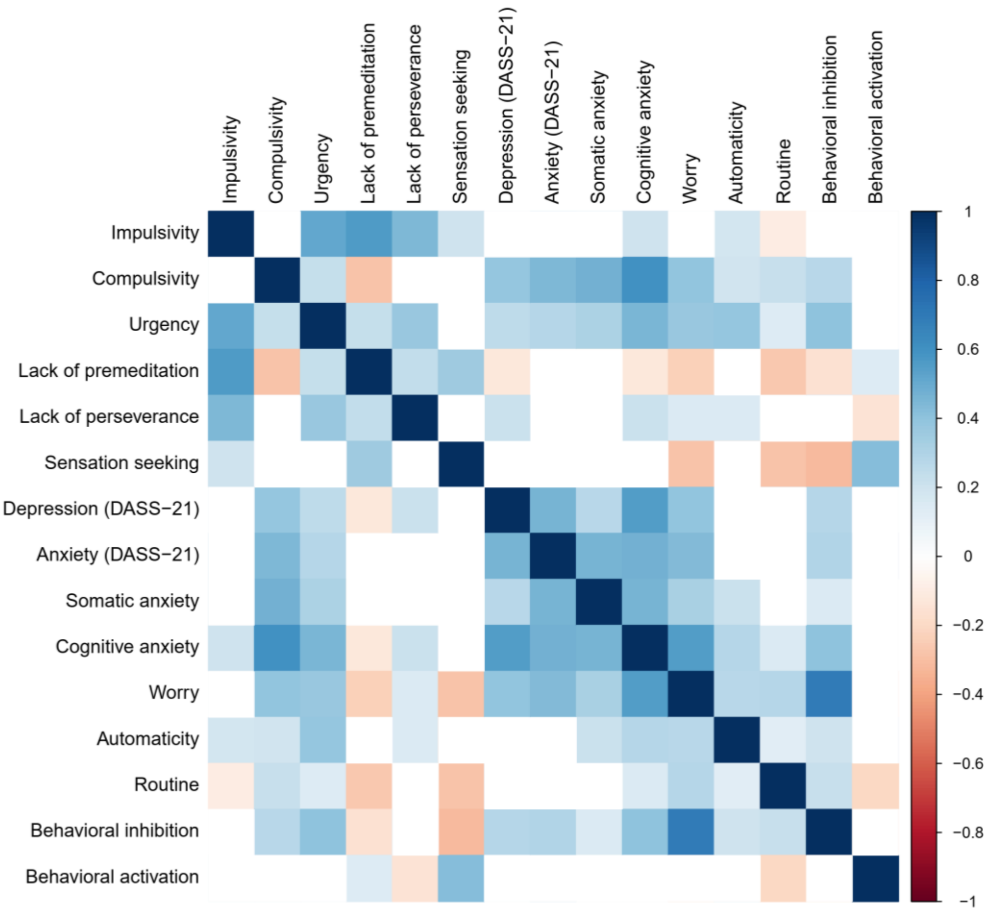

Heat map depicting all correlation coefficients (Spearman's correlations, two-tailed), p-values were corrected using the Bonferroni method ($p < 0.0004$), and white squares denote non-significant correlations (n = 221). Impulsivity and compulsivity: Barratt Impulsiveness Scale, revised [1]; Obsessive-Compulsive Inventory-Revised [4,5]; additional impulsivity facets: Urgency, Lack of premeditation, Lack of perseverance, Sensation seeking, Impulsive Behavior Scale [6-8]; Depression (DASS-21) and Anxiety (DASS-21): Depression Anxiety Stress Scales [9]; Somatic and Cognitive anxiety: State-Trait Inventory for Cognitive and Somatic Anxiety [11]; Worry: Penn State Worry Questionnaire [13]; Automaticity and Routine: subscales of habitual propensity measure Creature of Habit Scale [15,16]; Behavioral inhibition and activation: BIS/BAS scales [17,18].

Supplementary Table 2. Sample characteristics derived from the monetary incentive Flanker task: Measures of performance monitoring and task performance

	Mean	*SD*	*Range*
EEG (µV)			
ERN	-4.48	2.89	-15.29 – 2.64
ERN gain	-4.43	3.01	-15.44 – 2.89
ERN loss	-4.56	2.89	-15.17 – 2.36
CRN gain	-0.32	2.87	-13.91 – 8.13
CRN loss	-0.53	2.81	-8.39 – 8.30
Accuracy (%)			
Error rate	17.76	7.94	5.31 – 39.01
PEA	75.72	15.38	33.78 – 97.92
Reaction times (ms)			
PES	-1.90	14.93	-93.35 – 33.06
RT error	253.60	24.12	193.25 – 374.21
RT correct	375.35	31.74	286.00 – 510.29

ERN, error-related negativity; CRN, correct-related negativity; Error rate, percentage of errors; PEA, Post-error accuracy; RT, reaction time; PES, post-error slowing; Error reaction times refer to all incongruent error trials; Correct reaction times refer to incongruent correct trials.

Supplementary Table 3. Task characteristics regarding trial type for the monetary incentive Flanker task

	Mean	*SD*	*Range*
Trial type			
gain	318.67	4.99	256 – 320
loss	318.58	4.83	255 – 320
incongruent	318.38	5.03	255 – 320
congruent	318.88	4.80	256 – 320
Error trials			
incongruent	109.01	47.75	34 – 236
incongruent gain	58.57	25.01	16 – 123
incongruent loss	50.44	23.62	15 – 118
congruent	4.03	6.37	0 – 46
congruent gain	2.09	3.18	0 – 23
congruent loss	1.95	3.47	0 – 23
Correct trials			
incongruent	209.37	48.58	83 – 286
incongruent gain	100.68	25.35	36 – 144
incongruent loss	108.69	24.08	41 – 145
congruent	314.85	8.73	253 – 320
congruent gain	157.34	4.50	125 – 160
congruent loss	157.51	4.44	128 – 160

Descriptive statistics for trial numbers of the respective trial types in the Monetary Incentive Flanker task.

Supplementary Figure 2: Depiction of the error-related negativity and the correct-related negativity.

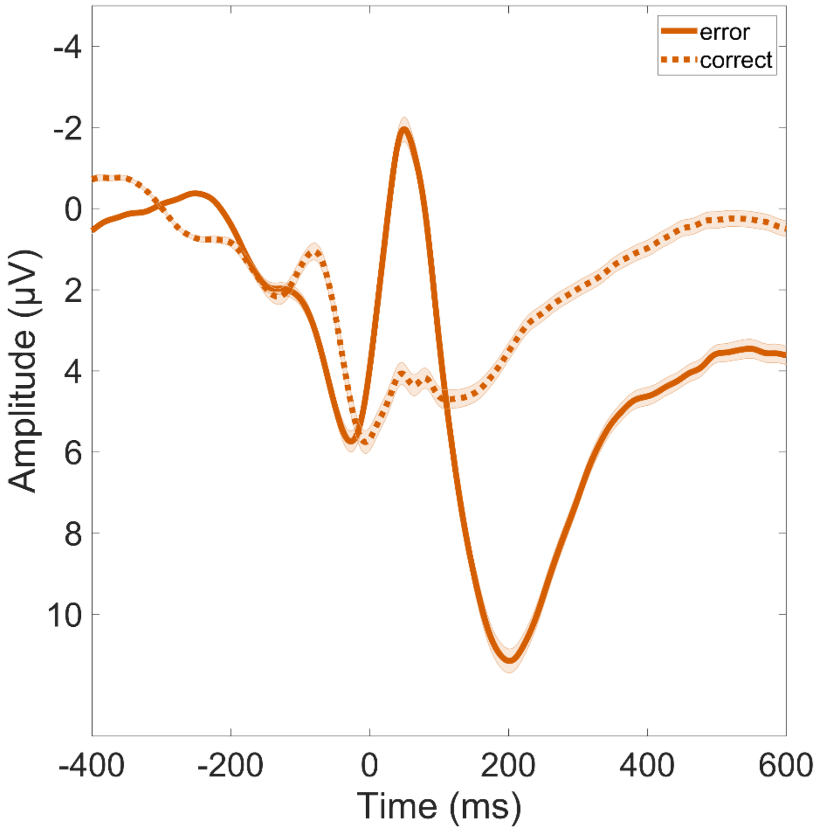

Time course of mean response-locked incongruent error and correct trials for the complete sample (n = 221) at Fz, FCz and Cz. Shades indicate the standard error of the mean (SEM).

Supplementary Notes 2: The effects of impulsivity and compulsivity on desire enactment

2.1 Predicting enactment. We fitted a logistic mixed-effects model to predict desire enactment with desire strength, conflict strength, impulsivity and compulsivity; and desire and conflict strength as random effects. Within this model, desire strength had a significant positive effect on enactment (β = 5.75, 95% CI [5.27, 6.24], p < .001; $\beta_{standardized}$ = 3.38, 95% CI [3.18, 3.58]), conflict strength had a significant negative effect (β = -1.83, 95% CI [-2.08, -1.57], p < .001; $\beta_{standardized}$ = -1.33, 95% CI [-1.46, -1.19]), impulsivity was not significant (β = -0.03, 95% CI [-0.77, 0.71], p = 0.938; $\beta_{standardized}$ = -0.02, 95% CI [-0.13, 0.09]) and compulsivity showed a significant positive effect (β = 0.31, 95% CI [0.06, 0.56], p = 0.016; $\beta_{standardized}$ = 0.11, 95% CI [0.01, 0.21]). Both interaction effects were significant (impulsivity and compulsivity with desire strength: β = -0.67, 95% CI [-1.12, -0.23], p = 0.003; $\beta_{standardized}$ = 0.03, 95% CI [-0.13, 0.18]; impulsivity and compulsivity with conflict strength: β = 0.41, 95% CI [0.18, 0.65], p < .001; $\beta_{standardized}$ = 0.02, 95% CI [-0.09, 0.13]). The model's total explanatory power was substantial (conditional R^2 = 0.78) and the part related to the fixed effects alone (marginal R^2) was 0.66. The model's intercept, corresponding to desire strength = 0, conflict strength = 0, impulsivity = 0, and compulsivity = 0, was at -3.49 (95% CI [-4.29, -2.68], p < .001).

Supplementary Table 4. Statistics for the full model analyzing the effect of impulsivity and compulsivity on desire enactment

	Enactment		
Predictors	*Odds Ratios*	*CI*	*p*
(Intercept)	0.03	0.01 – 0.07	**<0.001**
Desire strength	314.85	193.86 – 511.36	**<0.001**
Conflict strength	0.16	0.12 – 0.21	**<0.001**
Impulsivity	0.97	0.46 – 2.04	0.938
Compulsivity	1.36	1.06 – 1.75	**0.016**
Desire strength:Impulsivity:Compulsivity	0.51	0.32 – 0.80	**0.003**
Conflict strength:Impulsivity:Compulsivity	1.51	1.19 – 1.91	**0.001**
**Random Effects**			
σ^2	3.29		
$\tau_{00\ \text{Participant}}$	0.29		
$\tau_{11\ \text{Participant.Desire strength}}$	2.66		
$\tau_{11\ \text{Participant.Conflict strength}}$	0.73		
ρ_{01}	-0.59		
	0.35		
ICC	0.35		
$N_{\text{Participant}}$	221		
Observations	12353		
Marginal R^2 / Conditional R^2	0.660 / 0.778		

Impulsivity and compulsivity also significantly interacted with desire strength and conflict strength: in high impulsivity, high compulsivity led to an even weaker influence of desire and conflict strength on enactment. This was investigated using a simple slopes analysis.

For desire strength, the analysis revealed that, at low levels of impulsivity, the slope of desire strength was positive and significant across all levels of compulsivity (low compulsivity: β = 5.63, SE = 0.22, 95% CI [5.21, 6.05]; moderate compulsivity: β = 5.29, SE = 0.16, 95% CI [4.99, 5.60]; high compulsivity: β = 4.96, SE = 0.17, 95% CI [4.63, 5.29]). As impulsivity increased to moderate levels, the strength of the relationship between desire strength and enactment slightly weakened but remained significant, particularly at higher levels of compulsivity (low compulsivity: β = 5.61, SE = 0.21, 95% CI [5.19, 6.02]; moderate compulsivity: β = 5.22, SE = 0.15, 95% CI [4.92, 5.52]; high compulsivity: β = 4.82, SE = 0.20, 95% CI [4.44, 5.21]). At high levels of impulsivity, the strength of the relationship continued to weaken across all levels of compulsivity (low compulsivity: β = 5.59, SE = 0.206, 95% CI [5.18, 5.99]; moderate compulsivity: β = 5.14, SE = 0.153, 95% CI [4.84, 5.44]; high compulsivity: β = 4.69, SE = 0.227, 95% CI [4.24, 5.13]), but remained significant. These results suggest that desire strength is a stronger predictor of enactment when individuals score lower on impulsivity and compulsivity. For conflict strength, the analysis revealed that, at low levels of impulsivity, the association between conflict strength and enactment was negative and significant across all levels of compulsivity, with decreasing magnitude as

compulsivity increases (low compulsivity: β = -1.75, SE = 0.1128, 95% CI [-1.97, -1.53]; moderate compulsivity: β = -1.55, SE = 0.0813, 95% CI [-1.71, -1.39]; high compulsivity: β = -1.35, SE = 0.0868, 95% CI [-1.52, -1.18]). At moderate levels of impulsivity, the strength of the negative relationship between conflict strength and enactment decreased slightly but remained significant (low compulsivity: β = -1.74, SE = 0.1101, 95% CI [-1.96, -1.52]; moderate compulsivity: β = -1.50, SE = 0.0787, 95% CI [-1.66, -1.35]; high compulsivity: β = -1.26, SE = 0.0997, 95% CI [-1.46, -1.07]). At high levels of impulsivity, the negative association remained significant but weakened slightly (low compulsivity: β = -1.73, SE = 0.1075, 95% CI [-1.94, -1.52]; moderate compulsivity: β = -1.45, SE = 0.0785, 95% CI [-1.61, -1.30]; high compulsivity: β = -1.18, SE = 0.1163, 95% CI [-1.41, -0.95]). Slopes decreased in magnitude with increasing compulsivity. Therefore, there is evidence that both impulsivity and compulsivity lead to a weaker association between desire strength and enactment, as well as conflict strength and enactment.

2.2 Multiple regression for consistency with previous analyses. To establish consistency with previous analyses from our group [19] we additionally used an operationalization of self-control failures where the enactment of conflict-laden desires was divided by the number of questionnaires participants had responded to. Compulsivity also significantly predicted the proportion of enacted conflict-laden desires in all reported situations (β = 0.04, 95% CI [0.02, 0.07], $t(218) = 3.58$, $p < 0.001$, $\beta_{standardized}$ = 0.23, 95% CI [0.11, 0.36]), while impulsivity did not (β = 0.07, 95% CI [-0.03, 0.17], $t(218) = 1.40$, p = 0.164, $\beta_{standardized}$ = 0.09, 95% CI [-0.04, 0.22]). The regression model was significant (adjusted R^2 = 0.05, $F(2, 218) = 7.36$, $p < 0.001$) and an interaction between impulsivity and compulsivity yielded no additional explanatory effect. Our analyses therefore revealed evidence for high compulsivity leading to more self-control failures, confirming our initial hypothesis, while high impulsivity does not, against our expectations.

2.3 Predicting desire and conflict. We also fitted generalized linear models to predict desire occurrence, reported conflict and conflict strength with impulsivity and compulsivity. Compulsivity predicted desire occurrence (β = 0.07, 95% CI [0.02, 0.12], $t(218) = 2.98$, $p = 0.003$, $\beta_{standardized}$ = 0.20, 95% CI [0.07, 0.33]) and both conflict reporting (β = 0.05, 95% CI [0.02, 0.09], $t(218) = 2.88$, $p = 0.004$, $\beta_{standardized}$ = 0.19, 95% CI [0.06, 0.32]) and conflict strength (β = 0.22, 95% CI [0.09, 0.36], $t(218) = 3.19$, $p = 0.002$, $\beta_{standardized}$ = 0.21, 95% CI [0.08, 0.34]). There were no effects of impulsivity (desire: β = -0.05, 95% CI [-0.23, 0.13], $t(218) = -0.53$, $p = 0.597$, $\beta_{standardized}$ = -0.04, 95% CI [-0.17, 0.10]; conflict: β = 0.07, 95% CI [-0.08, 0.22], $t(218) = 0.95$, $p = 0.342$, $\beta_{standardized}$ = 0.06, 95% CI [-0.07, 0.19]; conflict strength: β = 0.14, 95% CI [-0.42, 0.69], $t(218) = 0.49$, $p = 0.626$, $\beta_{standardized}$ = 0.03, 95% CI [-0.10, 0.16]) and no additional explanatory effect of an interaction between impulsivity and compulsivity (desire occurrence: $\Delta F(1, 217) = 0.13$, $p = .723$; conflict occurrence: $\Delta F(1, 217) = 0.29$, $p = .589$; conflict strength: $\Delta F(1, 217) = 0.01$, $p = .948$). All regression models were statistically significant (desire: adjusted R^2 = 0.03, $F(2, 218) = 4.59$, $p = 0.011$; conflict: adjusted R^2 = 0.03, $F(2, 218) = 4.58$, $p = 0.011$; conflict strength: adjusted R^2 = 0.04, $F(2, 218) = 5.20$, $p = 0.006$). Thus, none of our hypotheses were confirmed: high compulsivity was associated with reporting more desires, conflict reporting and conflict strength, while impulsivity was not.

Supplementary Notes 3: The effects of impulsivity and compulsivity on the association between the ERN and desire enactment

3.1 Moderation analyses. As there is evidence for a connection between error-related brain activity and self-control [19,20] but also evidence for altered performance monitoring in disorders characterized by impulsivity and compulsivity, we wanted to investigate the moderating effect of these

two traits on the association between the ERN and self-control. We fitted logistic mixed-effects models to predict desire enactment with desire strength, conflict strength, the ERN, impulsivity and compulsivity. The models included desire and conflict strength as random effects. Within the base model, the effect of desire strength was statistically significant and positive (β = 5.22, 95% CI [4.91, 5.52], $p < .001$; $\beta_{standardized} = 3.38$, 95% CI [3.18, 3.58]). The effect of conflict strength was statistically significant and negative (β = -1.49, 95% CI [-1.65, -1.33], $p < .001$; $\beta_{standardized} = -1.32$, 95% CI [-1.46, -1.18]). The effect of the ERN was statistically non-significant and positive (β = 0.11, 95% CI [-0.08, 0.31], $p = 0.258$; $\beta_{standardized} = 0.06$, 95% CI [-0.05, 0.17]). The model's total explanatory power was substantial (conditional $R^2 = 0.78$) and the part related to the fixed effects alone (marginal R^2) was of 0.66. The model's intercept, corresponding to desire strength = 0, conflict strength = 0, and ERN = 0, was at -3.18 (95% CI [-3.41, -2.95], $p < .001$).

Supplementary Table 5. Statistics for the base model analyzing the effect of impulsivity and compulsivity on the association between the ERN and enactment

	Enactment		
Predictors	*Odds Ratios*	*CI*	*p*
(Intercept)	0.04	0.03 – 0.05	**<0.001**
Desire strength	184.77	136.30 – 250.48	**<0.001**
Conflict strength	0.22	0.19 – 0.26	**<0.001**
ERN	1.12	0.92 – 1.37	0.258
**Random Effects**			
σ^2	3.29		
τ_{00} Participant	0.32		
τ_{11} Participant.Desire_strength	2.80		
τ_{11} Participant.Conflict_strength	0.79		
ρ_{01}	-0.62		
	0.40		
ICC	0.36		
N Participant	221		
Observations	12353		
Marginal R^2 / Conditional R^2	0.656 / 0.779		

Within the full model including an interaction term with impulsivity and compulsivity, the effect of desire strength was statistically significant and positive (β = 5.22, 95% CI [4.91, 5.52], $p < .001$; $\beta_{standardized} = 3.38$, 95% CI [3.18, 3.58]). The effect of conflict strength was statistically significant and negative (β = -1.50, 95% CI [-1.65, -1.34], $p < .001$; $\beta_{standardized} = -1.32$, 95% CI [-1.46, -1.18]). Interestingly, the effect of the ERN was statistically significant and positive (β = 0.26, 95% CI [0.02, 0.51], $p = 0.033$; $\beta_{standardized} = 0.06$, 95% CI [-0.05, 0.17]). Finally, the interaction effect of the ERN with impulsivity and compulsivity was statistically significant and negative (β = -0.20, 95% CI [-0.38, -0.01], $p = 0.034$; $\beta_{standardized} = 0.01$, 95% CI [-0.12, 0.12]). The model's total explanatory power was substantial (conditional $R^2 = 0.78$) and the part related to the fixed effects alone (marginal R^2) was of 0.66. The

model's intercept, corresponding to desire strength = 0, conflict strength = 0, ERN = 0, impulsivity = 0 and compulsivity = 0, was at -3.18 (95% CI [-3.41, -2.95], $p < .001$).

Supplementary Table 6. Statistics for the full model analyzing the effect of impulsivity and compulsivity on the association between the ERN and enactment

	Enactment		
Predictors	*Odds Ratios*	*CI*	*p*
(Intercept)	0.04	0.03 – 0.05	**<0.001**
Desire strength	184.30	135.84 – 250.06	**<0.001**
Conflict strength	0.22	0.19 – 0.26	**<0.001**
ERN	1.30	1.02 – 1.66	**0.033**
ERN:Impulsivity:Compulsivity	0.82	0.68 – 0.99	**0.034**
**Random Effects**			
σ^2	3.29		
τ_{00} Participant	0.29		
τ_{11} Participant.Desire strength	2.83		
τ_{11} Participant.Conflict strength	0.79		
ρ_{01}	-0.61		
	0.37		
ICC	0.36		
N Participant	221		
Observations	12353		
Marginal R^2 / Conditional R^2	0.657 / 0.780		

3.2 Robustness analyses. To assess the robustness of fixed-effect estimates, we performed a parametric bootstrap analysis of the model. Using the fitted model, 2000 parametric bootstrap samples were generated with bootMer (*lme4* version 1.1-29 [21]). For each bootstrap sample, fixed effect parameters were re-estimated, and percentile-based 95% confidence intervals were computed. Bootstrap confidence intervals were compared with Wald-based estimates to evaluate the stability of parameter estimates (see Supplementary Table 7). To verify numerical stability of the three-way ERN:Impulsivity:Compulsivity interaction, we refitted the model using multiple optimizers via allFit (*lme4* version 1.1-29 [21]): bobyqua, Nelder_Mead, nlminbwrap, and nloptwrap variants. Across all convergent fits, the interaction estimate remained largely unchanged ($\beta \approx -0.20$). Minor convergence warnings in two nloptwrap fits (max|grad| ≈ 0.012-0.013) did not affect the estimate, indicating that the interaction is robust to the choice of optimizer. We additionally conducted a subset-perturbation sensitivity analysis; in each of the 30 iterations, 20 participants (~9% of the sample) were randomly removed and the model refit with the full random effects structure. Across all iterations, the interaction remained negative (mean β = -0.20, SD = 0.03, range = -0.27 to -0.14), indicating that the effect was not driven by a small set of participants.

Supplementary Table 7. Fixed-effect estimates from the generalized linear-mixed-effects model predicting enactment, with parametric bootstrap confidence intervals (2000 samples).

	Enactment			
Predictors	*Estimate (β)*	*CI*	*Odds Ratio*	*CI*
(Intercept)	-3.18	-3.43 – -2.95	0.04	0.03 – 0.05
Desire strength	5.22	4.93 – 5.51	184.30	138.54 – 247.38
Conflict strength	-1.50	-1.66 – -1.34	0.22	0.19 – 0.26
ERN	0.26	0.03 – 0.52	1.30	1.03 – 1.67
ERN:Impulsivity:Compulsivity	-0.20	-0.38 – -0.01	0.82	0.68 – 0.99

3.3 Simple slopes analyses. A simple slopes analysis was conducted to investigate the three-way interaction between the ERN, impulsivity and compulsivity. The results suggest that when both compulsivity and impulsivity are low, higher ERN amplitudes significantly predict lower probability of enactment. However, at higher levels of either impulsivity or compulsivity, the relationship between the ERN and enactment becomes non-significant.

The effect of impulsivity at different levels of compulsivity. Slopes for the effect of the ERN on enactment were calculated at low (-1 SD below the mean), moderate (mean), and high (+1 SD above the mean) levels of impulsivity. At low levels of compulsivity and low levels of impulsivity, the slope of the ERN was positive and significant, β = 0.23, SE = 0.11, 95% CI [0.01, 0.45], indicating that as the ERN decreased (indicated by more positive values), the amount of enactments significantly increased. The slope remained positive but marginally non-significant at low compulsivity and moderate levels of impulsivity, β = 0.22, SE = 0.11, 95% CI [−0.01, 0.44], and at low compulsivity and high levels of impulsivity, β = 0.21, SE = 0.11, 95% CI [−0.01, 0.43]. At moderate levels of compulsivity, the slopes of the ERN were non-significant across all levels of impulsivity: At low impulsivity, the slope was β = 0.13, SE = 0.10, 95% CI [−0.07, 0.33]; at moderate impulsivity, β = 0.10, SE = 0.10, 95% CI [−0.09, 0.30]; and at high impulsivity, β = 0.08, SE = 0.10, 95% CI [−0.12, 0.28]. This was also true at high levels of compulsivity: At low impulsivity, the slope was β = 0.03, SE = 0.11, 95% CI [−0.18, 0.24]; at moderate impulsivity, β = −0.01, SE = 0.12, 95% CI [−0.24, 0.22]; and at high impulsivity, β = −0.05, SE = 0.13, 95% CI [−0.30, 0.20].

The effect of compulsivity at different levels of impulsivity. Slopes for the effect of the ERN on enactment were calculated at low (-1 SD below the mean), moderate (mean), and high (+1 SD above the mean) levels of compulsivity. At low levels of impulsivity and low levels of compulsivity, the slope of the ERN was positive and significant, β = 0.23, SE = 0.11, 95% CI [0.01, 0.45], indicating that as the ERN decreased (indicated by more positive values), the amount of enactments significantly increased. The slope was non-significant at moderate (β = 0.13, SE = 0.10, 95% CI [−0.07, 0.33]) and high levels of compulsivity (β = 0.03, SE = 0.11, 95% CI [−0.18, 0.24]). At moderate levels of impulsivity, the slopes of the ERN were non-significant across all levels of compulsivity: At low compulsivity, the slope was β = 0.22, SE = 0.11, 95% CI [−0.01, 0.44]; at moderate compulsivity, β = 0.10, SE = 0.10, 95% CI [−0.09, 0.30]; and at high levels, β = −0.01, SE = 0.12, 95% CI [−0.24, 0.22]. At high levels of impulsivity, this was also true: all slopes were non-significant: At low compulsivity, β = 0.21, SE = 0.11, 95% CI [−0.01, 0.43]; at

moderate compulsivity, β = 0.08, SE = 0.10, 95% CI [−0.12, 0.28]; at high compulsivity β = −0.05, SE = 0.13, 95% CI [−0.30, 0.20].

3.4 No difference between motivational contexts. Results did not differ between gain and loss contexts. To analyze the gain context, we fitted a logistic mixed model (estimated using ML and BOBYQA optimizer) to predict enactment with desire strength, conflict strength, ERN gain, Impulsivity and Compulsivity (formula: enactment ~ desire strength + conflict strength + ERN gain + ERN gain:impulsivity:compulsivity). The model included desire and conflict strength as random effects (formula: ~1 + desire strength + conflict strength | participant). The model's total explanatory power was substantial (conditional R^2 = 0.78) and the part related to the fixed effects alone (marginal R^2) was of 0.66. The model's intercept, corresponding to desire strength = 0, conflict strength = 0, ERN gain = 0, impulsivity = 0 and compulsivity = 0, is at -3.20 (95% CI [-3.43, -2.97], p < .001). Within this model, the effect of desire strength was statistically significant and positive (β = 5.22, 95% CI [4.91, 5.52], p < .001; $\beta_{standardized}$ = 3.38, 95% CI [3.18, 3.58]); the effect of conflict strength was statistically significant and negative (β = -1.50, 95% CI [-1.65, -1.34], p < .001; $\beta_{standardized}$ = -1.32, 95% CI [-1.46, -1.18]); the effect of ERN gain was statistically non-significant and positive (β = 0.23, 95% CI [-6.98e-03, 0.47], p = 0.057; $\beta_{standardized}$ = 0.05, 95% CI [-0.06, 0.16]); the interaction effect of ERN gain with impulsivity and compulsivity was statistically significant and negative (β = -0.19, 95% CI [-0.38, -4.58e-03], p = 0.045; $\beta_{standardized}$ = 0.02, 95% CI [-0.11, 0.14]). Standardized parameters were obtained by fitting the model on a standardized version of the dataset. 95% Confidence Intervals (CIs) and p-values were computed using a Wald z-distribution approximation.

Supplementary Table 8. Statistics for the full model analyzing the effect of impulsivity and compulsivity on the association between the ERN in the gain context and enactment

	Enactment		
Predictors	*Odds Ratios*	*CI*	*p*
(Intercept)	0.04	0.03 – 0.05	**<0.001**
Desire strength	184.42	135.93 – 250.22	**<0.001**
Conflict strength	0.22	0.19 – 0.26	**<0.001**
ERN gain	1.26	0.99 – 1.60	0.057
ERN gain:Impulsivity:Compulsivity	0.82	0.68 – 1.00	**0.045**
**Random Effects**			
σ^2	3.29		
τ_{00} Participant	0.29		
τ_{11} Participant.Desire strength	2.83		
τ_{11} Participant.Conflict strength	0.79		
ρ_{01}	-0.61		
	0.37		
ICC	0.36		
N Participant	221		
Observations	12353		
Marginal R^2 / Conditional R^2	0.657 / 0.780		

To analyze the loss context, we fitted a logistic mixed model (estimated using ML and nlminbwrap optimizer) to predict enactment with desire strength, conflict strength, ERN gain, Impulsivity and Compulsivity (formula: enactment ~ desire strength + conflict strength + ERN loss + ERN loss:impulsivity:compulsivity). The model included desire and conflict strength as random effects (formula: ~1 + desire strength + conflict strength | participant). The model's total explanatory power was substantial (conditional $R^2 = 0.78$) and the part related to the fixed effects alone (marginal R^2) was of 0.66. The model's intercept, corresponding to desire strength = 0, conflict strength = 0, ERN gain = 0, impulsivity = 0 and compulsivity = 0, was at -3.17 (95% CI [-3.40, -2.94], $p < .001$). Within this model, the effect of desire strength was statistically significant and positive ($\beta = 5.22$, 95% CI [4.91, 5.52], $p < .001$; $\beta_{standardized} = 3.38$, 95% CI [3.18, 3.58]); the effect of conflict strength was statistically significant and negative ($\beta = -1.50$, 95% CI [-1.65, -1.34], $p < .001$; $\beta_{standardized} = -1.32$, 95% CI [-1.46, -1.18]); the effect of ERN loss is statistically significant and positive ($\beta = 0.27$, 95% CI [0.03, 0.52], $p = 0.027$; $\beta_{standardized} = 0.06$, 95% CI [-0.05, 0.17]); the interaction effect of ERN loss with impulsivity and compulsivity was statistically significant and negative ($\beta = -0.20$, 95% CI [-0.38, -0.02], $p = 0.030$; $\beta_{standardized} = -0.02$, 95% CI [-0.13, 0.10]). Standardized parameters were obtained by fitting the model on a standardized version of the dataset. 95% Confidence Intervals (CIs) and p-values were computed using a Wald z-distribution approximation.

Supplementary Table 9. Statistics for the full model analyzing the effect of impulsivity and compulsivity on the association between the ERN in the loss context and enactment

	Enactment		
Predictors	*Odds Ratios*	*CI*	*p*
(Intercept)	0.04	0.03 – 0.05	**<0.001**
Desire strength	184.18	135.76 – 249.89	**<0.001**
Conflict strength	0.22	0.19 – 0.26	**<0.001**
ERN loss	1.32	1.03 – 1.68	**0.027**
ERN loss:Impulsivity:Compulsivity	0.82	0.68 – 0.98	**0.030**
**Random Effects**			
σ^2	3.29		
$\tau_{00\ \text{Participant}}$	0.29		
$\tau_{11\ \text{Participant.Desire strength}}$	2.82		
$\tau_{11\ \text{Participant.Conflict strength}}$	0.79		
ρ_{01}	-0.61		
	0.37		
ICC	0.36		
$N_{\text{Participant}}$	221		
Observations	12353		
Marginal R^2 / Conditional R^2	0.657 / 0.780		

Both high impulsivity and compulsivity are therefore associated with a diminished association between self-control failures and the ERN, independent of motivational context (hypotheses partly confirmed). They also interact in moderating the association between ERN and self-control failures, and this association is attenuated when impulsivity and compulsivity are high.

3.5 Multiple regression for consistency with previous analyses. The results were similar when investigating the association between ERN and the proportion of enacted conflict-laden desires in all reported situations. In the base model (self-control failures ~ ERN), the effect of the ERN was not significant (β = 0.02, 95% CI [-0.00, 0.05], *t*(219) = 1.69, *p* = 0.092, $\beta_{standardized}$ = 0.11, 95% CI [-0.02, 0.25]; the regression model was not significant with an adjusted R^2 = 0.01, F(1, 219) = 2.87, *p* = 0.09). The ERN significantly predicted self-control in daily life (β = 0.06, 95% CI [0.03, 0.09], *t*(218) = 3.78, *p* < 0.001, $\beta_{standardized}$ = 0.30, 95% CI [0.14, 0.45]) only when including an interaction with impulsivity and compulsivity (β = -0.05, 95% CI [-0.08, -0.03], *t*(218) = -4.10, *p* < 0.001, $\beta_{standardized}$ = -0.32, 95% CI [-0.48, -0.17]). The regression model (self-control failures ~ ERN + ERN:impulsivity:compulsivity) was significant (adjusted R^2 = 0.08, F(2, 218) = 9.93, *p* < 0.001). Simple slopes analyses were performed to investigate this further. The slopes of the ERN were examined at three levels of compulsivity (low, mean, high) across three levels of Imp (low: -1 SD below the mean, moderate: mean, and high: +1 SD above the mean). When impulsivity was low, the relationship between the ERN and enactment varied across levels of compulsivity: at low compulsivity, there was a significant positive slope for the ERN predicting self-control failures, β = 0.05, SE = 0.02, t(218) = 3.45, *p* = .001; at mean compulsivity, the slope was positive and significant, β = 0.03, SE = 0.01, t(218) = 1.99, *p* = .048.; at high compulsivity, the slope was small and not significant, β = 0.01, SE = 0.01, t(218) = 0.08, *p* = .934. When impulsivity was moderate, the slopes of the ERN also varied: at low compulsivity), the slope was positive and significant, β = 0.05, SE = 0.01, t(218) = 3.39, *p* = .001; at mean compulsivity, the slope was not significant, β = 0.02, SE = 0.01, t(218) = 1.54, *p* = .123; at high compulsivity, the slope was near zero and not significant, β = -0.01, SE = 0.02, t(218) = -0.60, *p* = .550. When impulsivity was high, the relationship between the ERN and enactment weakened further: at low compulsivity, the slope was positive and significant, β = 0.05, SE = 0.01, t(218) = 3.32, *p* = .001.; at mean compulsivity the slope was small and not significant, β = 0.01, SE = 0.01, t(218) = 1.08, *p* = .28; at high compulsivity the slope was negative and not significant, β = -0.02, SE = 0.02, t(218) = -1.16, *p* = .27. The analysis revealed that the relationship between the ERN and enactment is moderated by both impulsivity and compulsivity. At low levels of impulsivity, there is a significant positive association between the ERN and enactment when compulsivity is low, suggesting that the ERN may have more influence on self-control failures. However, as impulsivity increases, the strength of this relationship diminishes, particularly at higher levels of compulsivity, where the slopes become non-significant or close to zero. This indicates that the combined effect of high impulsivity and compulsivity may reduce the impact of ERN on self-control in daily life.

Supplementary Figure 3: The moderating effects of impulsivity and compulsivity on the association of ERN and self-control failures

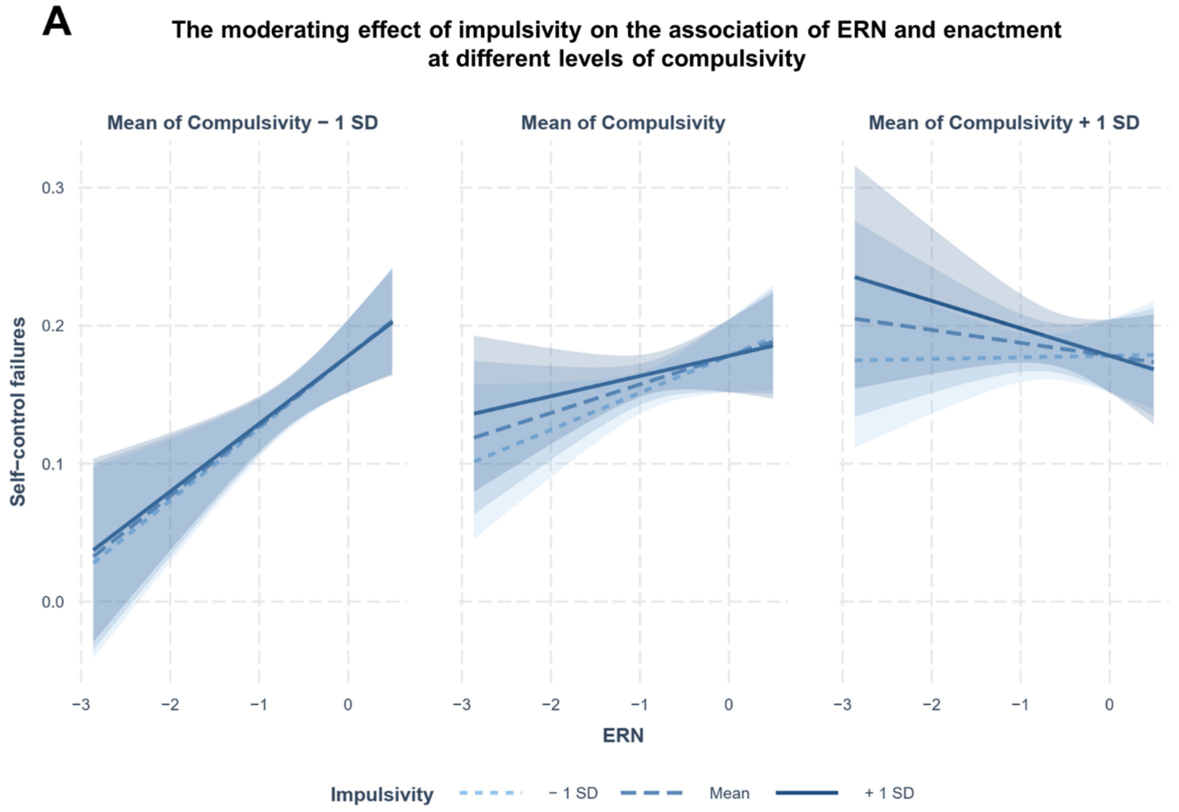

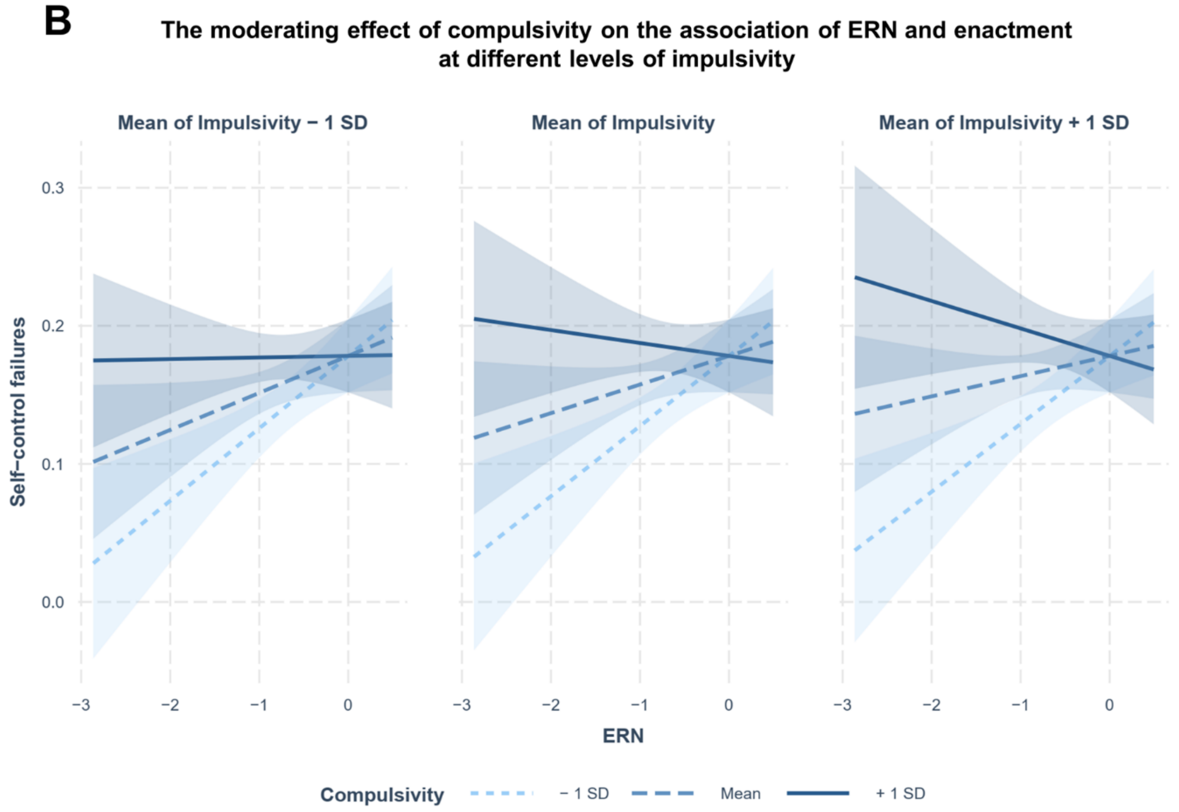

Visualization of the moderating effects of impulsivity and compulsivity on the association between the ERN amplitude and self-control failures (n = 221). This association was attenuated with higher impulsivity and compulsivity. **A** Simple slopes for the moderating effect of impulsivity on the association between the ERN and self-control failures at different levels of compulsivity. First panel, simple slopes for the association between ERN amplitude and self-control failures for low (-1 SD below the mean), moderate (mean), and high (+1 SD above the mean) levels of impulsivity, in low (-1 SD below the mean) compulsivity. Second panel, simple slopes for the association between ERN amplitude and self-control failures for low (-1 SD below the mean), moderate (mean), and high (+1 SD above the mean) levels of impulsivity, in moderate (mean) compulsivity. Third panel, simple slopes for the association between ERN amplitude and self-control failures for low (-1 SD below the mean), moderate (mean), and high (+1 SD above the mean) levels of impulsivity, in high (+1 SD above the mean) compulsivity. **B** Simple slopes for the moderating effect of compulsivity on the association between the ERN and self-control failures at different levels of impulsivity. First panel, simple slopes for the association between ERN amplitude and self-control failures for low (-1 SD below the mean), moderate (mean), and high (+1 SD above the mean) levels of compulsivity, in low (-1 SD below the mean) impulsivity. Second panel, simple slopes for the association between ERN amplitude and self-control failures for low (-1 SD below the mean), moderate (mean), and high (+1 SD above the mean) levels of compulsivity, in moderate (mean) impulsivity. Third panel, simple slopes for the association between ERN amplitude and self-control failures for low (-1 SD below the mean), moderate (mean), and high (+1 SD above the mean) levels of compulsivity, in high (+1 SD above the mean) impulsivity.

Supplementary Notes 4: Additional analyses regarding the correct-related negativity and behavioral parameters

4.1 Additional analyses regarding event-related potentials. The CRN was neither significantly associated with self-control in the gain context (β = 0.01, 95% CI [-0.01, 0.02], $t(219) = 0.76$, $p = 0.451$, $\beta_{standardized} = 0.05$, 95% CI [-0.08, 0.19]; model: adjusted $R^2 = -0.00$, $F(1, 219) = 0.64$, $p = 0.423$), nor the loss context (β = 0.00, 95% CI [-0.01, 0.02], $t(219) = 0.33$, p = 0. 740, $\beta_{standardized} = 0.02$, 95% CI [-0.11, 0.16]; model: adjusted $R^2 = -0.00$, $F(1, 219) = 0.12$, $p = 0.729$). This was also true for the early Pe (gain: β = 0.00, 95% CI [-0.02, 0.03], $t(219) = 0.19$, $p = 0.852$, $\beta_{standardized} = 0.01$, % CI [-0.12, 0.15]; model: adjusted $R^2 = -0.00$, $F(1, 219) = 0.03$, $p = 0.852$; loss: β = 0.00, 95% CI [-0.02, 0.03], $t(219) = 0.28$, $p = 0.777$, $\beta_{standardized} = 0.02$, 95% CI [-0.11, 0.15]; model: adjusted $R^2 = -0.00$, $F(1, 219) = 0.08$, $p = 0.777$) and the late Pe (gain: β = 0.01, 95% CI [-0.03, 0.04], $t(219) = 0.29$, $p = 0.770$, $\beta_{standardized} = 0.02$, 95% CI [-0.11, 0.15]; model: adjusted $R^2 = -0.00$, $F(1, 219) = 0.09$, $p = 0.770$; loss: β = 0.00, 95% CI [-0.03, 0.04], $t(219) = 0.02$, $p = 0.983$, $\beta_{standardized} = 0.00$, 95% CI [-0.13, 0.13]; model: adjusted $R^2 = -0.00$, $F(1, 219) = 0.00$, $p = 0.983$).

4.2 Additional analyses regarding behavioral parameters. Post-error slowing (β = 0.01, 95% CI [-0.01, 0.02], $t(219) = 0.37$, $p = 0.713$, $\beta_{standardized} = 0.02$, 95% CI [-0.11, 0.16]; model: adjusted $R^2 = -0.00$, $F(1, 219) = 0.14$, $p = 0.713$), the reaction time (RT) on correct incongruent (β = -0.05, 95% CI [-0.23, 0.13], $t(219) = -0.56$, $p = 0.575$, $\beta_{standardized} = -0.04$, 95% CI [-0.17, 0.10]; model: adjusted $R^2 = -0.00$, $F(1, 219) = 0.32$, $p = 0.575$) and the RT on erroneous incongruent trials (β = -0.12, 95% CI [-0.27, 0.04], $t(219) = -1.45$, $p = 0.148$, $\beta_{standardized} = -0.10$, 95% CI [-0.23, 0.03]; model: adjusted $R^2 = 0.01$, $F(1, 219) = 2.11$, p = 0. 148) showed no significant association with self-control (all $p > 0.1$). However, higher error rate was associated with lower self-control (β = 0.06, 95% CI [0.02, 0.10], $t(219) = 3.24$, $p = 0.001$, $\beta_{standardized} = 0.21$, 95% CI [0.08, 0.34]; model: adjusted $R^2 = 0.04$, $F(1, 219) = 10.49$, $p = 0.001$), higher post-error accuracy (β = -0.14, 95% CI [-0.21, -0.07], $p < 0.001$, $\beta_{standardized} = -0.25$, 95% CI [-0.38, -0.12]; model: adjusted $R^2 = 0.06$, $F(1, 219) = 14.12$, $p < 0.001$), and higher post-correct accuracy (β = -0.20, 95% CI [-

0.33, -0.07], $p = 0.003$, $\beta_{standardized} = 0.11$, 95% CI [0.01, 0.12]; model: adjusted $R^2 = 0.04$, $F(1, 219) = 9.16$, $p = 0.003$) predicted higher self-control in daily life.

Supplementary Notes 5: Descriptive statistics on desire type and conflict

5.1 Descriptive statistics. Desires were assessed in 18 main categories, including eating, drinking, drinking alcohol, smoking, using other substances, using the internet, playing computer games or gaming, watching TV or streaming, buying something, gambling, exercising, sleeping, resting, retreating, misbehaving, socializing, sex or intimacy, using the bathroom or personal hygiene. The most frequently reported desires were everyday activities, including eating (n = 2244 occurrences), using the bathroom which included personal hygiene (n = 737), resting (n = 732), drinking (n = 725), and sleeping (n = 704). Focusing on conflict-laden desires, the highest number of reported conflicts occurred for routine behaviors: eating (n = 697 conflicts), resting (n = 475), sleeping (n = 418), using the internet (n = 306), and watching TV or streaming (n = 213). Average conflict strength was highest for desires that more strongly involve self-control or social regulation rather than physiological needs: misbehaving (mean = 4.53, SD = 1.36), gambling (mean = 4.45, SD = 1.37), socializing (mean = 4.22, SD = 1.51), resting (mean = 4.18, SD = 1.40), and sleeping (mean = 4.15, SD = 1.40).

Supplementary Notes 6: Additional analyses for the Profile regression

6.1 Sensitivity analyses. A sensitivity analysis was conducted to evaluate the robustness of the identified cluster-level risk profiles under alternative distributional assumptions. Supplementary Table 10 summarizes the posterior means and 90% credible intervals for the low- and high-risk clusters across three model specifications: the primary (standard) model, a model assuming independent normal covariate distributions within clusters, and a model with a separation prior on the within-cluster covariance matrix.

Supplementary Table 10. Posterior means across different model specifications

Model	*Mean Low*	*90% CI Low*	*Mean High*	*90% CI High*
Standard	0.149	0.123 – 0.174	0.178	0.149 – 0.208
Independent Normal	0.147	0.111 – 0.184	0.167	0.142 – 0.192
Separation Prior	0.159	0.134 – 0.184	0.165	0.128 – 0.202

The posterior difference in risk between clusters remained small across all models (Standard: Mean Δ = 0.030, 90% CI [-0.007, 0.067]; Independent Normal: Mean Δ = 0.020, 90% CI [-0.025, 0.064]; Separation Prior: Mean Δ = 0.007, 90% CI [-0.038, 0.051]), indicating that conclusions were not driven by particular prior or covariance structure assumptions and results are robust. Notably, the uncertainty in cluster separation was comparable across all models, suggesting that it reflects inherent variability in the data rather than sensitivity to modeling choices.

6.2 Additional moderation analyses. The ERN predicted the proportion of enacted conflict-laden desires relative to all reported desires as a composite measure of self-control failures ($\beta = 0.04$, 95% CI [0.01, 0.07], $t(218) = 2.73$, $p = 0.007$, $\beta_{standardized} = 0.20$, 95% CI [0.06, 0.35]) in a model (adjusted R^2 = 0.04, $F(2, 218) = 5.04$, $p = 0.007$), that also revealed a significant interaction effect between the ERN and cluster membership ($\beta = -0.04$, 95% CI [-0.07, -0.01], $t(218) = -2.67$, $p = 0.008$, $\beta_{standardized} = -0.20$, 95% CI [-0.34, -0.05]).

Additionally, in a follow-up moderation analysis, the ERN also predicted the composite measure of self-control failures (β = 0.11, 95% CI [0.01, 0.21], *t*(217) = 2.11, *p* = 0.036, $\beta_{standardized}$ = 0.53, 95% CI [0.04, 1.03]; model: adjusted R^2 = 0.04, F(3, 217) = 5.04, *p* = 0.005). A three-way interaction between ERN, cluster membership and impulsivity was significant (β = -0.07, 95% CI [-0.21, -0.01], *t*(217) = -2.14, *p* = 0.034, $\beta_{standardized}$ = -0.54, 95% CI [-1.03, -0.04]), indicating that high impulsivity attenuated the association between the ERN and self-control failures within the cluster defined by high compulsivity, anxiety and worrying. This modulation was not significant in the cluster defined by low compulsivity, anxiety and worrying (β = -0.07, 95% CI [-0.16, 0.03], *t*(217) = -1.35, *p* = 0.177, $\beta_{standardized}$ = -0.36, 95% CI [-0.88, 0.16]).

Supplementary Figure 4: The moderating effects of cluster membership and impulsivity on the association of ERN and self-control failures

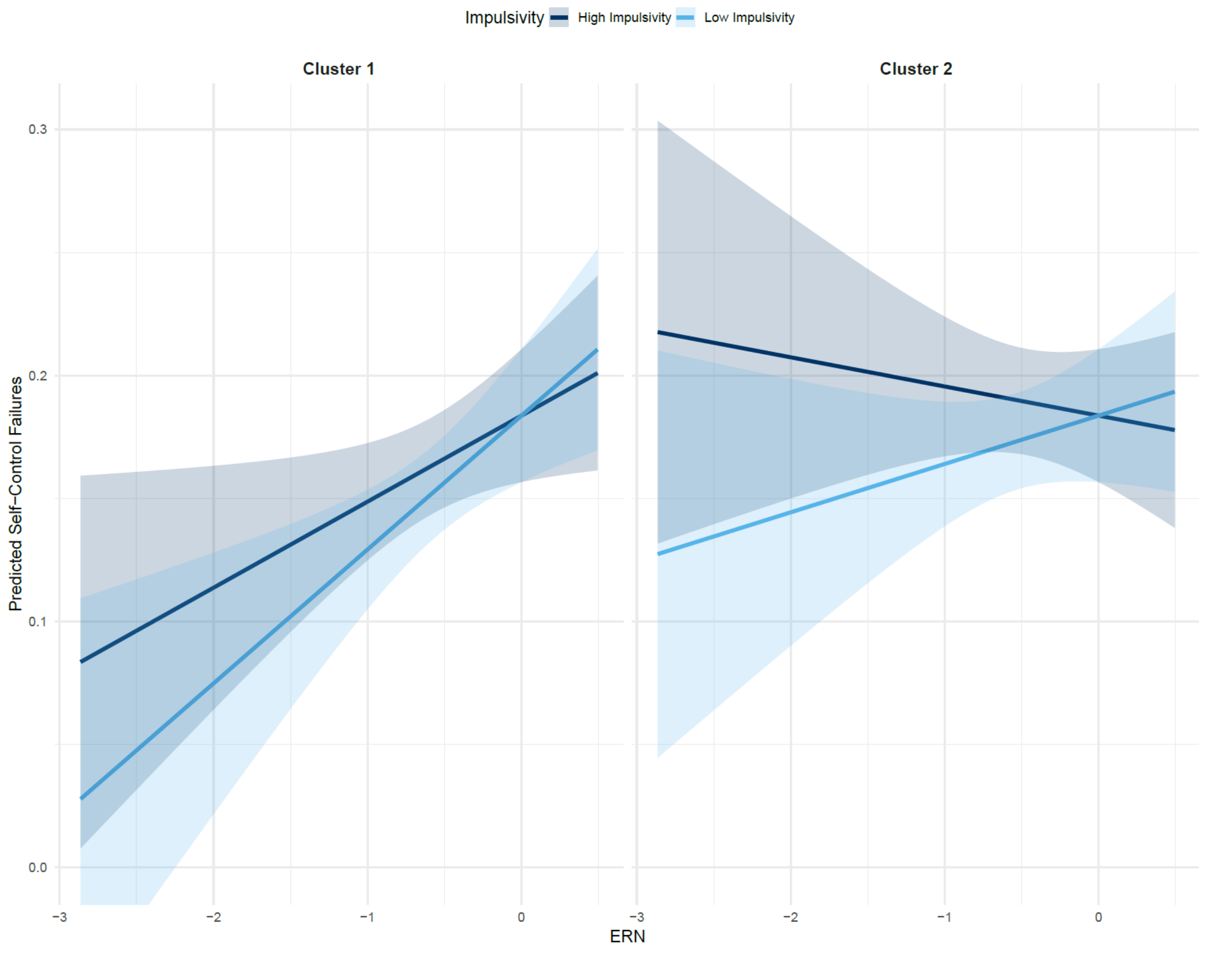

Visualization of the moderating effects of cluster membership and impulsivity on the association between the ERN amplitude and self-control failures (n = 221). This association was attenuated within cluster 2, which was defined by high compulsivity, anxiety and worrying. Shown are simple slopes for the moderating effect of impulsivity on the association between the ERN and self-control failures within cluster 1, defined by low compulsivity, anxiety and worrying, and cluster 2, defined by high compulsivity, anxiety and worrying. First panel, simple slopes for the association between ERN amplitude and self-control failures for low (-1 SD below the mean) and high levels (+1 SD below the mean) of impulsivity, in cluster 1. Second panel, simple slopes for the association between ERN amplitude and self-control failures for low (-1 SD below the mean) and high levels (+1 SD below the mean) of impulsivity, in cluster 2.

Supplementary References

1 Patton, J. H., Stanford, M. S. & Barratt, E. S. Factor structure of the Barratt impulsiveness scale. *Journal of clinical psychology* **51**, 768-774, doi:10.1002/1097-4679(199511)51:6<768::AID-JCLP2270510607>3.0.CO;2-1 (1995).

2 Stanford, M. S. *et al.* Fifty years of the Barratt Impulsiveness Scale: An update and review. *Personality and individual differences* **47**, 385-395, doi:10.1016/j.paid.2009.04.008 (2009).

3 Dück, K., Overmeyer, R., Eger, L. & Endrass, T. Self-Reported Impulsivity Predicts Missed Study Appointments: Validating a German Adaptation of the BIS-11. *Personality Science* **6**, 27000710251408750, doi:10.1177/27000710251408750 (2025).

4 Foa, E. B. *et al.* The Obsessive-Compulsive Inventory: development and validation of a short version. *Psychological assessment* **14**, 485, doi:10.1037/1040-3590.14.4.485 (2002).

5 Gönner, S., Leonhart, R. & Ecker, W. The German version of the obsessive-compulsive inventory-revised: a brief self-report measure for the multidimensional assessment of obsessive-compulsive symptoms. *Psychotherapie, Psychosomatik, Medizinische Psychologie* **57**, 395-404, doi:10.1055/s-2007-970894 (2007).

6 Schmidt, R. E., Gay, P., d'Acremont, M. & Van der Linden, M. A German Adaptation of the UPPS Impulsive Behavior Scale: Psychometric Properties and Factor Structure. *Swiss Journal of Psychology* **67**, 107-112, doi:10.1024/1421-0185.67.2.107 (2008).

7 Whiteside, S. P. & Lynam, D. R. The five factor model and impulsivity: Using a structural model of personality to understand impulsivity. *Personality and individual differences* **30**, 669-689, doi:10.1016/S0191-8869(00)00064-7 (2001).

8 Whiteside, S. P., Lynam, D. R., Miller, J. D. & Reynolds, S. K. Validation of the UPPS impulsive behaviour scale: a four-factor model of impulsivity. *European Journal of personality* **19**, 559-574, doi:10.1002/per.556 (2005).

9 Henry, J. D. & Crawford, J. R. The short-form version of the Depression Anxiety Stress Scales (DASS-21): Construct validity and normative data in a large non-clinical sample. *British journal of clinical psychology* **44**, 227-239, doi:10.1348/014466505X29657 (2005).

10 Nilges, P. & Essau, C. Die depressions-angst-stress-skalen. *Der Schmerz* **29**, 649-657, doi:10.1007/s00482-015-0019-z (2015).

11 Ree, M. J., French, D., MacLeod, C. & Locke, V. Distinguishing cognitive and somatic dimensions of state and trait anxiety: Development and validation of the State-Trait Inventory for Cognitive and Somatic Anxiety (STICSA). *Behavioural and Cognitive Psychotherapy* **36**, 313-332, doi:10.1017/S1352465808004232 (2008).

12 Overmeyer, R. & Endrass, T. J. C. P. i. E. Cognitive Symptoms Link Anxiety and Depression Within a Validation of the German State-Trait Inventory for Cognitive and Somatic Anxiety (STICSA). **5**, doi:10.32872/cpe.9753 (2023).

13 Stöber, J. Besorgnis: ein vergleich dreier inventare zur erfassung allgemeiner sorgen. *Zeitschrift für differentielle und diagnostische Psychologie* **16**, 50-63 (1995).

14 Molina, S. & Borkovec, T. D. The Penn State Worry Questionnaire: Psychometric properties and associated characteristics. (1994).

15 Ersche, K. D., Lim, T.-V., Ward, L. H., Robbins, T. W. & Stochl, J. Creature of Habit: A self-report measure of habitual routines and automatic tendencies in everyday life. *Personality and Individual Differences* **116**, 73-85, doi:10.1016/j.paid.2017.04.024 (2017).

16 Overmeyer, R., Fürtjes, S., Ersche, K. D., Ehrlich, S. & Endrass, T. Self-regulation is negatively associated with habit tendencies: A validation of the German Creature of Habit Scale. *Personality and Individual Differences* **163**, 110029, doi:10.1016/j.paid.2020.110029 (2020).

17 Strobel, A., Beauducel, A., Debener, S. & Brocke, B. Eine deutschsprachige version des BIS/BAS-Fragebogens von carver und white. *Zeitschrift für Differentielle und diagnostische Psychologie*, doi:10.1024/0170-1789.22.3.216 (2001).

18 Carver, C. S. & White, T. L. Behavioral inhibition, behavioral activation, and affective responses to impending reward and punishment: the BIS/BAS scales. *Journal of personality and social psychology* **67**, 319, doi:10.1037/0022-3514.67.2.319 (1994).
19 Overmeyer, R. *et al.* The error-related negativity predicts self-control failures in daily life. *Frontiers in Human Neuroscience* **14**, 614979, doi:10.3389/fnhum.2020.614979 (2021).
20 Krönke, K.-M. *et al.* Monitor yourself! Deficient error-related brain activity predicts real-life self-control failures. *Cognitive, Affective, & Behavioral Neuroscience* **18**, 622-637, doi:10.3758/s13415-018-0593-5 (2018).
21 Bates, D. *et al.* Package 'lme4'. *convergence* **12**, 2 (2015).
