# Supplementary material for: The association between the error-related negativity and self-control is moderated by impulsivity and compulsivity
Source: Commun Psychol. 2026 Mar 27;4:62. doi: 10.1038/s44271-026-00446-3 (PMC13046855; doi:10.1038/s44271-026-00446-3)
Supplement: Supplementary file 3 — Reporting Summary [file 44271_2026_446_MOESM3_ESM.pdf]

nature portfolio

Corresponding author(s): Rebecca Overmeyer

Last updated by author(s): Mar 2, 2026

Reporting Summary

Nature Portfolio wishes to improve the reproducibility of the work that we publish. This form provides structure for consistency and transparency in reporting. For further information on Nature Portfolio policies, see our Editorial Policies and the Editorial Policy Checklist.

Statistics

For all statistical analyses, confirm that the following items are present in the figure legend, table legend, main text, or Methods section.

n/a	Confirmed	
☐	☒	The exact sample size (*n*) for each experimental group/condition, given as a discrete number and unit of measurement
☐	☒	A statement on whether measurements were taken from distinct samples or whether the same sample was measured repeatedly
☐	☒	The statistical test(s) used AND whether they are one- or two-sided<br>*Only common tests should be described solely by name; describe more complex techniques in the Methods section.*
☐	☒	A description of all covariates tested
☐	☒	A description of any assumptions or corrections, such as tests of normality and adjustment for multiple comparisons
☐	☒	A full description of the statistical parameters including central tendency (e.g. means) or other basic estimates (e.g. regression coefficient) AND variation (e.g. standard deviation) or associated estimates of uncertainty (e.g. confidence intervals)
☒	☐	For null hypothesis testing, the test statistic (e.g. *F*, *t*, *r*) with confidence intervals, effect sizes, degrees of freedom and *P* value noted<br>*Give P values as exact values whenever suitable.*
☐	☒	For Bayesian analysis, information on the choice of priors and Markov chain Monte Carlo settings
☐	☒	For hierarchical and complex designs, identification of the appropriate level for tests and full reporting of outcomes
☐	☒	Estimates of effect sizes (e.g. Cohen's *d*, Pearson's *r*), indicating how they were calculated

Our web collection on statistics for biologists contains articles on many of the points above.

Software and code

Policy information about availability of computer code

Data collection	The Flanker task was presented using Presentation 19.0 (Neurobehavioral Systems Inc., Berkeley, CA, USA). EMA was delivered using an EMA application (movisensXS, version 1.3.3; movisens GmbH, Karlsruhe, Germany). EEG preprocessing was performed using EEGLAB and MATLAB 2018b (The MathWorks Inc., 2018).
Data analysis	All statistical analyses were performed using R [70]. Hierarchical linear models were fitted using the R package lme4 version 1.1-29, general linear models were fitted using the R package stats, and assumptions were checked using the R package DHARMa version 0.4.6. Simple slopes were tested using the emmeans R package version 1.8.3. Cluster analysis was performed using the R packages cluster version 2.1.6, factoextra version 1.0.7, and the PReMiuM package version 3.2.13 in R.<br>Data and code for the analyses are available on the OSF (https://doi.org/10.17605/OSF.IO/4GPND).

For manuscripts utilizing custom algorithms or software that are central to the research but not yet described in published literature, software must be made available to editors and reviewers. We strongly encourage code deposition in a community repository (e.g. GitHub). See the Nature Portfolio guidelines for submitting code & software for further information.

Data

Policy information about availability of data

All manuscripts must include a data availability statement. This statement should provide the following information, where applicable:

- Accession codes, unique identifiers, or web links for publicly available datasets
- A description of any restrictions on data availability
- For clinical datasets or third party data, please ensure that the statement adheres to our policy

The data and code that support the findings of this study are available in the Open Science Framework with the identifier [https://doi.org/10.17605/OSF.IO/4GPND].

Research involving human participants, their data, or biological material

Policy information about studies with human participants or human data. See also policy information about sex, gender (identity/presentation), and sexual orientation and race, ethnicity and racism.

Reporting on sex and gender	Participants self-reported their sex, assessed via the questionnaire item 'biologisches Geschlecht' ('biological sex'). No separate item assessed gender identity. Due to ambiguity in the German term 'Geschlecht', which can refer to both sex and gender, misclassification cannot be entirely ruled out.<br>Sex was considered in the study design to reduce sampling bias, with recruitment stratified to ensure a balanced sample (46.6% female). However, sex was not included in statistical analyses, as the study focused on transdiagnostic traits (impulsivity and compulsivity) in relation to self-control, independent of sex or gender.<br>Disaggregated self-reported sex data are available in the source data file. All participants consented to the sharing of anonymized individual-level data.
Reporting on race, ethnicity, or other socially relevant groupings	We collected three socially relevant self-report variables to characterize the sample: (1) highest educational attainment, (2) history of mental health problems, and (3) self-ascribed ancestry.<br>Ancestry was assessed through self-report, with an option to describe ancestry in an open field. Of the final sample, 93.7% (n = 207) had completed higher education, 11.3% reported a history of mental health problems, 91.8% identified as primarily of European ancestry, 5.9% as Asian or Middle Eastern, and 2.3% as unknown.<br>These variables were collected exclusively via self-report and used only to assess sample representativeness. None were included as predictors or covariates in any statistical model. We did not use ancestry as a proxy for race, ethnicity, or cultural identity. No socially constructed variables were included in our main or exploratory analyses, and no such variables were statistically controlled for.<br>In the German research context, questions about race, ethnicity, and other socially defined identities have traditionally been approached with caution due to the historical legacy of racial classification and persecution under the National Socialist regime. This context informed our decision to use such variables in a limited and purely descriptive manner. We acknowledge, however, that scientific discourse in Germany is evolving—as is our own practice.
Population characteristics	See above.
Recruitment	Participants were recruited through online and offline advertisements targeting individuals with varying levels of impulsivity and compulsivity, as assessed via short screening questionnaires. This recruitment strategy aimed to capture a broad range along these transdiagnostic dimensions, rather than a representative general population sample. Participants volunteered to take part in a multi-method study that included EEG assessment and ecological momentary assessment (EMA) of daily self-control behavior.<br>As with any volunteer-based study, self-selection bias is possible. Individuals with higher interest in psychology or personal insight into their behavior may have been more likely to participate. Moreover, the relatively high proportion of participants with higher education may limit generalizability. However, because the primary focus was on within-sample associations between individual difference variables (e.g., impulsivity, compulsivity, self-control), and not on population-level prevalence estimates, the potential impact of self-selection on the main results is likely limited.
Ethics oversight	All procedures complied with the ethical guidelines of the Declaration of Helsinki and were approved by the Ethics Committee at the Technische Universität Dresden (EK 372092017). All participants provided written informed consent.

Note that full information on the approval of the study protocol must also be provided in the manuscript.

Field-specific reporting

Please select the one below that is the best fit for your research. If you are not sure, read the appropriate sections before making your selection.

☐ Life sciences ☒ Behavioural & social sciences ☐ Ecological, evolutionary & environmental sciences

For a reference copy of the document with all sections, see nature.com/documents/nr-reporting-summary-flat.pdf

Behavioural & social sciences study design

All studies must disclose on these points even when the disclosure is negative.

Study description	The study is a quantitative, observational design combining laboratory-based EEG measures, behavioral task data, self-report questionnaires, and ecological momentary assessment (EMA) of self-control in daily life.
Research sample	The research sample consisted of community volunteers recruited in and around Dresden, Germany. Participants were selected to represent a broad range along the dimensions of impulsivity and compulsivity, based on screening questionnaires, rather than to form a representative sample of the general population. The final sample included 221 participants (46.6% female; 25.16 years, SD = 4.94, rage = 18-45 years). Most participants (93.7%) had completed higher education degrees.<br>This sampling strategy was chosen to investigate transdiagnostic traits across a wide variability spectrum, which is critical for studying dimensional relationships between impulsivity, compulsivity, and self-control. The goal was not to generalize to the general population, but to examine trait-based associations in a diverse, non-clinical sample.
Sampling strategy	Participants were recruited using a convenience sampling procedure with targeted screening: Individuals were invited through online and offline advertisements and selected to ensure a broad range along the dimensions of impulsivity and compulsivity, based on brief self-report screening measures.<br>Sample size was determined a priori based on power calculations for detecting small effects ($f^2 = 0.05$) in linear multiple regression models with three predictors, using G*Power. A minimum sample of N = 223 was estimated to achieve 80% power at $\alpha = .05$. To account for potential data loss (e.g., EEG data quality, noncompliance with EMA, attrition), a larger sample of N = 253 was recruited.
Data collection	Data were collected in a two-part procedure: (1) a laboratory session including EEG recording and a computerized cognitive task (Monetary Incentive Flanker Task), and (2) a 7-day ecological momentary assessment (EMA) period using smartphones provided by the research team.<br>EEG was recorded using elastic EEG caps with 64 Ag/AgCl electrodes and BrainAmp amplifiers (Brain Products GmbH, Munich, Germany). The cognitive task was presented using Presentation software on a lab computer. For EMA, participants carried identical Nokia 5 smartphones with the app movisensXS (version 1.3.3; movisens GmbH, Karlsruhe, Germany), which delivered 8 signal-contingent questionnaires per day.<br>During the lab session, only the participant and trained study personnel were present. During the EMA period, participants completed the assessments independently in their daily environments. While the lead researchers were not blind to the study hypotheses, most study personnel involved in data collection were blind to the hypotheses. Experimental conditions in the Flanker task (gain vs. loss context) were fully counterbalanced and randomized within subjects.
Timing	Data collection began in April 2018 and was completed in September 2019.
Data exclusions	Data were excluded based on pre-established criteria defined in the preregistration. Specifically, participants were excluded if they had: (1) an overall error rate > 40% in the cognitive task, (2) fewer than 16 artifact-free error trials per condition for EEG analysis, (3) evidence of noncompliance with task instructions (e.g., random button presses or excessive responses outside the valid response window), (4) poor EEG data quality (e.g., ICA did not converge), or (5) missing EMA data (did not respond to any EMA questionnaires).<br>Based on these criteria, we excluded 32 participants after data collection for the following reasons: poor EEG data quality (n = 1), discontinuation of assessment (n = 1), not following task instructions as indicated by excessive random button presses during the task (n = 7), fewer than 16 errors (n = 11) per motivational context [66], and an error rate exceeding 40 % (n = 12).
Non-participation	A total of 280 participants were enrolled and included in the study following the application of exclusion criteria. Of these, 27 participants dropped out after inclusion and consent. Documented reasons for dropout included lack of time (n = 14), relocation (n = 4), loss of interest (n = 4), and job-related incompatibility with the EMA procedure (n = 5). The final sample consisted of 253 participants.
Randomization	Participants were not allocated into experimental groups.

Reporting for specific materials, systems and methods

We require information from authors about some types of materials, experimental systems and methods used in many studies. Here, indicate whether each material, system or method listed is relevant to your study. If you are not sure if a list item applies to your research, read the appropriate section before selecting a response.

Materials & experimental systems

n/a	Involved in the study
☒	☐ Antibodies
☒	☐ Eukaryotic cell lines
☒	☐ Palaeontology and archaeology
☒	☐ Animals and other organisms
☒	☐ Clinical data
☒	☐ Dual use research of concern
☒	☐ Plants

Methods

n/a	Involved in the study
☒	☐ ChIP-seq
☒	☐ Flow cytometry
☒	☐ MRI-based neuroimaging

Plants

Seed stocks

Report on the source of all seed stocks or other plant material used. If applicable, state the seed stock centre and catalogue number. If plant specimens were collected from the field, describe the collection location, date and sampling procedures.

Novel plant genotypes

Describe the methods by which all novel plant genotypes were produced. This includes those generated by transgenic approaches, gene editing, chemical/radiation-based mutagenesis and hybridization. For transgenic lines, describe the transformation method, the number of independent lines analyzed and the generation upon which experiments were performed. For gene-edited lines, describe the editor used, the endogenous sequence targeted for editing, the targeting guide RNA sequence (if applicable) and how the editor was applied.

Authentication

Describe any authentication procedures for each seed stock used or novel genotype generated. Describe any experiments used to assess the effect of a mutation and, where applicable, how potential secondary effects (e.g. second site T-DNA insertions, mosiacism, off-target gene editing) were examined.
